# Artificial Intelligence-Based Application Provides Accurate Medical Triage Advice When Compared to Consensus Decisions of Healthcare Providers

**DOI:** 10.7759/cureus.16956

**Published:** 2021-08-06

**Authors:** Sean Delshad, Venkata S Dontaraju, Vipindas Chengat

**Affiliations:** 1 Internal Medicine, David Geffen School of Medicine at University of California Los Angeles, Los Angeles, USA; 2 Internal Medicine, Javon Bea Hospital, Rockford, USA; 3 Internal Medicine, Mountainview Medical Center, Las Vegas, USA

**Keywords:** artificial intelligence in medicine, triage, misdiagnosis, clinical decision support system, clinical variability, value in healthcare, healthcare application, healthcare expenditure

## Abstract

Accurate medical triage is essential for improving patient outcomes and efficient healthcare delivery. Patients increasingly rely on artificial intelligence (AI)-based applications to access healthcare information, including medical triage advice. We assessed the accuracy of triage decisions provided by an AI-based application. We presented 50 clinical vignettes to the AI-based application, seven emergency medicine providers, and five internal medicine physicians. We compared the triage decisions of the AI-based application to those of the individual providers as well as their consensus decisions. When compared to the human clinicians’ consensus triage decisions, the AI-based application performed equal or better than individual human clinicians.

## Introduction

Timely and accurate triage of various medical conditions is vital to improving patient outcomes and the efficiency of healthcare delivery systems [1,2]. Inaccurate triage may lead to process delays and low-value care. As an example, up to two-thirds of emergency department visits are unnecessary and avoidable, resulting in $32 billion in excess national healthcare expenditure [3].

Artificial intelligence (AI) has tremendous potential to improve healthcare, including medical triage optimization [4-6]. Machine learning algorithms utilized in the emergency department have already been shown to better predict clinical outcomes than conventional methods [7]. However, a significant proportion of triage occurs even prior to the emergency room visit and is determined by the patient. Patients currently utilize search engines, chatbots, and AI-based personal health assistant applications to access medical information that may aid in initial triage decisions, underscoring the importance of these services’ accuracy.

Prior research indicates that online symptom checkers often provide inaccurate or incomplete diagnostic or triage advice for users [8,9]. One audit study found that appropriate triage advice was given only in 57% of standardized patient evaluations [10]. More recent research demonstrated that an AI-based virtual assistant appropriately triaged 90% of clinical vignettes presented to it [11]. However, this determination of accuracy was made by one human clinician, significantly limiting the strength of this finding.

We sought to measure the accuracy of triage decisions provided by another AI-based application, MayaMD, by directly comparing its performance to physicians and physician assistants of multiple medical specialties practicing in various clinical settings.

## Materials and methods

MayaMD is an AI-based application that patients may utilize to help determine where they should seek care for any medical condition. Using this application, patients input symptoms and answer subsequent questions, and are then provided with likely diagnoses as well as whether to continue with self-care or seek primary, urgent, or emergency care services.

MayaMD uses a combination of Bayesian statistics and pattern recognition. Layers of supervised and unsupervised machine learning sit on top of a core algorithm to recognize new patterns, typically resulting from changing geographic or demographic data. MayaMD’s library and core algorithm are built on accepted evidence-based clinical knowledge and include over 7,000 diagnoses, 8,500 initial inputs (symptoms, physical signs, and labs), 40,000 inferences, and 2,200 medications and interactions. Similar AI-based applications have also been previously described [11-13].

**Figure 1:**
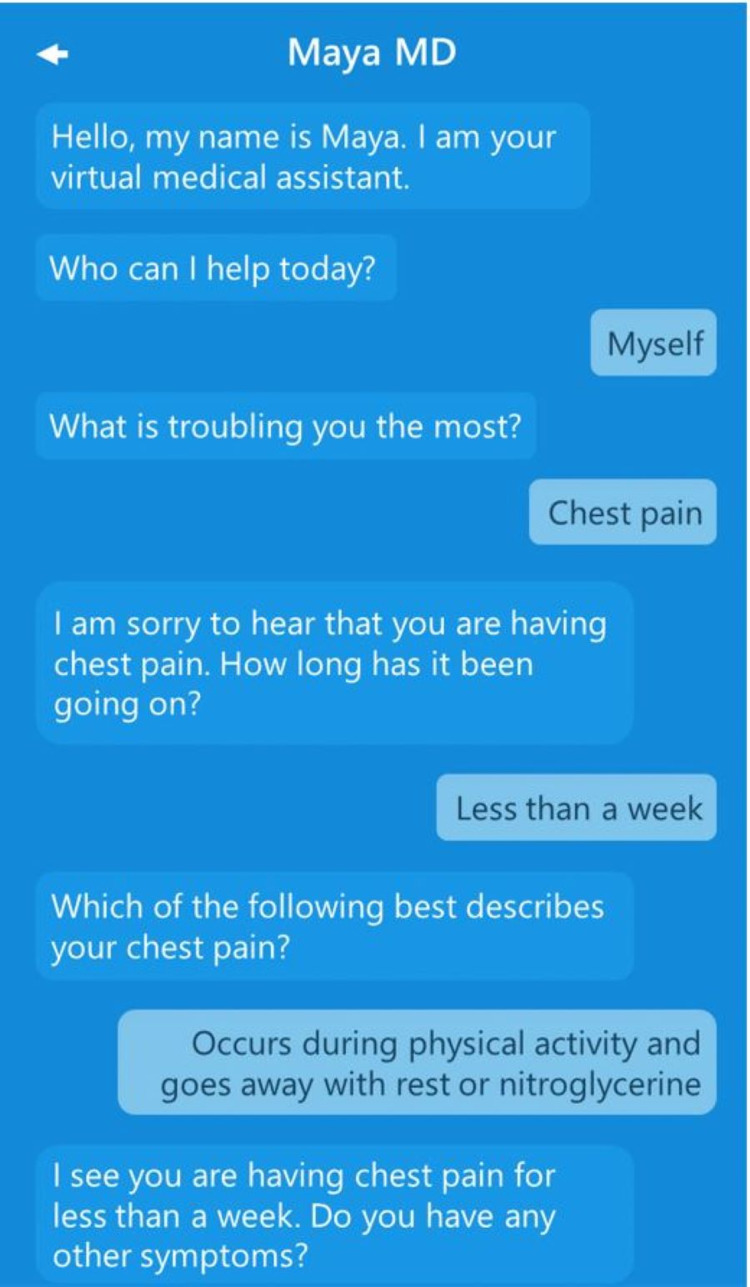
Example of a user interacting with MayaMD.

MayaMD guides the user to answer various questions that ultimately serve as inputs for the algorithm to recommend optimal triage. The individual inputs symptoms and their quality, duration, and alleviating and exacerbating factors. MayaMD also asks the user about various risk factors associated with the input symptoms. For example, if the user states they are having chest pain, MayaMD will ask if the user has ever smoked or had a blot clot in the legs. Figure 1 demonstrates an example of a user interacting with the MayaMD application. 

The MayaMD algorithm utilizes these inputs to determine a differential diagnosis that also guides the optimal triage decision. Any life-threatening condition (based on symptoms, risk factors, and differential diagnosis) is triaged to the emergency room. Any case that requires immediate intervention, such as antibiotic or nebulized breathing treatment, without pre-determined “red flags” that may indicate a life-threatening condition is triaged to urgent care. Chronic symptoms that MayaMD does not associate with any life-threatening differential diagnoses are triaged to outpatient services.

We sought to measure the accuracy of triage decisions provided by MayaMD. We compared triage decisions made by the AI-based application with those made by healthcare providers for various medical conditions. We simulated patients presenting with various medical conditions by developing 50 unique clinical vignettes, similar to prior studies [9,11]. The vignettes were selected from a list of the most common clinical presentations to the emergency room, urgent care, and primary care services. Vignettes included the patient’s age, sex, medical history, and presenting symptoms with a variable amount of pertinent positive and negative symptoms. Vignettes were not associated with a definite diagnosis or expected standard of care. Vignettes were not associated with an available physical examination. An example of one of the vignettes utilized in the study is as follows: "80-year-old man with a history of coronary artery disease presented with shortness of breath for 2-3 weeks. He complains of waking up in the middle of the night, gasping for air. He also feels uncomfortable sleeping flat, without pillows. All the symptoms started a few weeks ago." The full list of clinical vignettes is presented as supplementary material (see Appendices).

Providers were recruited in three phases. In the first phase, the Department of Bioinformatics at the University of Utah assisted in recruiting emergency room (ER) providers who participated in the study. For the second and third phases, physicians were recruited via the American Medical Association’s Physician Innovation Network, where a posting advertised a survey opportunity for cash. For the second phase, six physicians from the University of Michigan St. Joseph Mercy Hospital Emergency Medicine Residency Program participated in the study. For phase three, five internal medicine hospitalists practicing at the University of California Los Angeles (UCLA) Health participated in the study.

In all three phases, providers were asked to read each vignette and then choose one of the following options for each vignette: (1) “Go to ER or call 911 (life-threatening injuries or symptoms that need treatment immediately)”; (2) “Go to urgent care within 24 hours (non-life-threatening, but need treatment the same day)”; (3) “Go to primary care physician (PCP) within three days (not immediately life-threatening that can wait three days before being seen by a primary care physician or specialist)”; or (4) “Self-care, remain at home, and only report to primary care or urgent care if the condition worsens.” MayaMD provides one of these four triage decisions for patients based on the information they input into the application.

In the first phase of the study, we compared triage decisions between an ER physician, ER physician assistant, and MayaMD for 50 unique clinical vignettes. We recorded MayaMD and each provider’s triage decision for each vignette.

In the second phase of the study, we selected only the vignettes from the first phase of the study in which the two providers and MayaMD did not provide the same triage decision. These vignettes were then presented to six ER physicians practicing at the University of Michigan St. Joseph Mercy Hospital. Again, we recorded triage decisions by each physician. All six physicians were then asked to discuss each vignette and develop a consensus triage decision for each clinical vignette.

In the third phase of the study, we presented all 50 clinical vignettes to five internal medicine physicians practicing hospitalist medicine at UCLA Health. Each physician provided a triage decision, and then the group discussed the cases to develop a consensus triage decision for each vignette. In the second and third phases of the study, individual provider’s and MayaMD’s triage decisions were then compared with consensus triage decisions.

## Results

In the first phase of the study, we compared triage decisions between an ER physician, ER physician assistant, and MayaMD for 50 clinical vignettes. The ER physician and MayaMD agreed on triage decisions in 37 (74%) cases, while the ER physician assistant and MayaMD agreed on 30 (60%) cases. There was significant variability in the ER physician and ER physician assistant's responses, who agreed on triage decisions for only 28 (56%) cases. The ER physician, ER physician assistant, and MayaMD all agreed on triage decisions in only 24 cases (48%).

For the second phase of the study, we selected 26 vignettes from the first phase of the study that did not result in a unanimous triage decision and presented these vignettes to six other ER physicians practicing at a different health system. The six physicians agreed on the same triage decision for nine (35%) cases. The physicians were then asked to come to a consensus triage decision for all 26 cases, hereby referred to as “Consensus A.” MayaMD made the same triage decision as the physicians in Consensus A for 22 (85%) cases. The individual ER physicians had the same triage decision as the decision in Consensus A for an average of 21 (82%) cases, with a range from 18 to 24 (69% to 92%) cases. Only one of the physicians matched Consensus A decisions more times than MayaMD matched Consensus A decisions.

Utilizing Consensus A decisions and the 24 unanimous decisions from phase one of the study, we developed a second consensus of triage decisions, hereby referred to as “Consensus B.” When compared to Consensus B triage decisions, the ER physician from phase one of the study, ER physician assistant from phase one of the study, and MayaMD made the same decision for 39 (78%), 30 (60%), and 46 (92%) clinical vignettes, respectively.

In the third phase of the study, we again presented all 50 clinical vignettes to six other providers, and then asked them to reach a consensus for each vignette, hereby referred to as “Consensus C.” On average, an individual physician’s initial triage decisions matched Consensus C decisions for 40 cases (80%), with a range from 31 to 45 (62% to 90%) cases. MayaMD came to the same triage decision as the Consensus C decision for 44 (88%) cases.

The triage decisions of MayaMD and Consensuses A, B, and C for all 50 vignettes are presented in Table 1. The decisions of individual providers for all 50 vignettes are presented as a table in the Appendices.

**Table 1:** Triage decisions of MayaMD and consensuses. E - Emergency room U - Urgent care P - Primary care S - Self-care

Case	MayaMD Decision	Consensus B Decision (includes Consensus A)	Consensus C Decision
1	E	E	E
2	S	S	S
3	U	U	U
4	P	P	P
5	P	P	P
6	P	P	P
7	E	E	U
8	E	E	E
9	S	S	S
10	P	P	P
11	P	P	P
12	P	P	P
13	E	E	E
14	E	E	E
15	S	S	S
16	P	E	P
17	P	P	P
18	E	E	E
19	E	E	E
20	E	E	E
21	P	P	P
22	E	E	E
23	E	E	E
24	E	E	E
25	U	E	U
26	E	E	U
27	E	E	E
28	E	E	E
29	P	P	P
30	P	P	P
31	E	E	E
32	E	E	E
33	P	P	P
34	U	U	U
35	E	E	U
36	P	P	P
37	P	P	U
38	P	P	P
39	P	P	P
40	E	E	E
41	P	S	P
42	U	U	E
43	P	P	P
44	U	U	U
45	P	P	P
46	E	E	E
47	P	P	P
48	U	E	E
49	P	P	P
50	E	E	E

## Discussion

**Figure 2:**
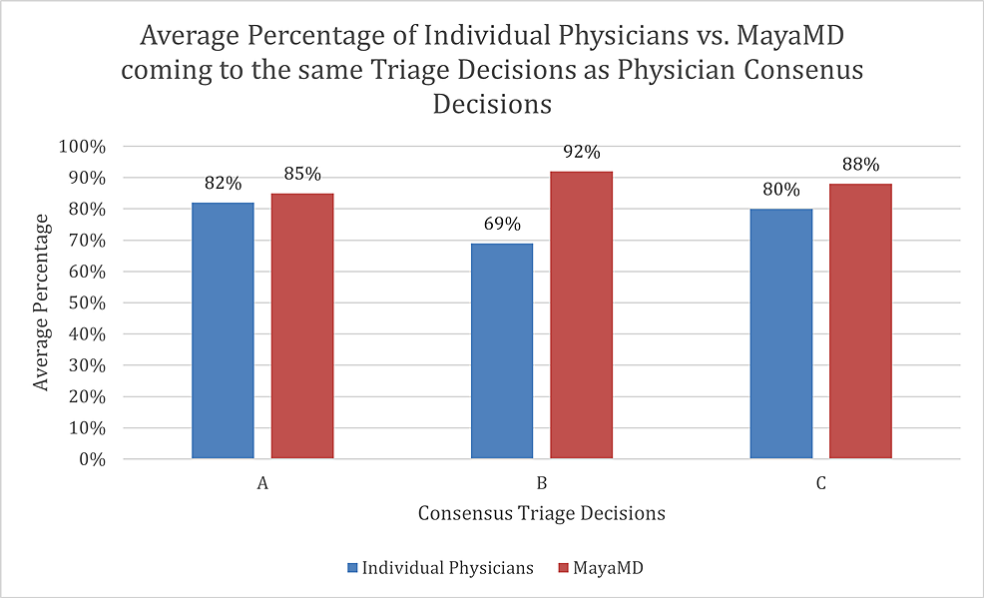
Average percentage of individual physicians vs. MayaMD coming to the same triage decisions as physician consensus decisions.

Our results demonstrate that an AI-based application, MayaMD, performs at par or better than individual clinicians when determining a triage decision for a clinical vignette (Figure 2). In our study, the AI-based application’s triage decisions accurately matched the triage decisions of two consensuses of six practicing human physicians. The AI-based application’s decisions were consistent with the consensus decisions at a similar rate to the individual human physicians’ decisions consistency with the consensus decisions.

Among the 50 clinical vignettes, there was a total of nine cases in which the AI-based application came to a different decision than at least one of the consensus groups. There were four cases (16, 25, 41, 48) where MayaMD had a different decision than Consensus B and six cases (7, 26, 35, 37, 42, 48) where MayaMD had a different decision than Consensus C. In six of these cases (7, 25, 26, 35, 42, 48), the discrepancy was due to triage to urgent versus emergency care services. There were similar differences in the decisions of individual providers in these cases, demonstrating that either triage may be appropriate. This discrepancy may also be due to individual biases based on practice patterns at various urgent care facilities, as there is substantial variability in what medical conditions are managed at urgent care facilities throughout the United States [14]. For Case 16, a 45-year-old man with a history of heavy smoking presenting with leg pain, the AI-based application recommended primary care services, whereas Consensus B recommended emergency care services. Consensus B likely came to this decision to account for the differential diagnosis of critical limb ischemia; however, based on the other details of the case, both the AI-based application and Consensus C recommended primary care services. Similar discrepancies were found for Cases 37 and 41. Based on these cases, it is clear that minimizing variability in the triage decision relies on detailed input from the user to help narrow the differential diagnosis associated with the user’s symptoms, especially to reduce the likelihood of diagnoses that are potentially life-threatening. Interestingly, there was only one case (48) in which both consensus groups came to a different decision than the AI-based application, and in this case, the discrepancy was between urgent versus emergency care services. Other than this one case, the AI-based application’s triage decision was consistent with at least one of the consensus groups’ decisions, demonstrating the validity of the AI-based application as a medical triage tool.

Our study builds upon previous research that has demonstrated that AI-based applications can determine appropriate diagnoses and appropriately triage for various clinical presentations. While earlier research on the accuracy and safety of various symptom checkers provided mixed and unfavorable results [8-10], more recent research examining AI-based applications for this purpose has been more positive. For example, Barriga et al. demonstrated an AI-based application was able to accurately predict discharge diagnosis from patient’s input of symptoms upon arriving at the emergency room [15]. Another study indicated that an AI-based virtual assistant appropriately triaged 90% of clinical vignettes presented to it, and Gilbert et al. demonstrated that three available AI-based applications provided safe triage advice when compared to general practitioners for 200 clinical vignettes [11,16].

Our study is the first to our knowledge to compare AI triage decisions with those of a consensus of a group of providers from multiple specialties working at various practice locations, including both academic and non-academic settings. Our findings are significantly strengthened by this utilization of a consensus group of diverse, actively practicing clinicians. Our study is also the first to our knowledge that demonstrates the ability of AI to discriminate between ER and urgent care triage, as previous studies only assessed the AI-based application’s ability to determine urgent versus non-urgent triage. Appropriate triage discrimination between urgent and emergency care services has significant implications for cost savings and efficient healthcare delivery for health insurers and health systems.

Throughout the various phases of the study, there was high variability among the individual providers' decisions, similar to previous research [12]. In our study, ER providers generally agreed on clinical presentations that required emergency care but had more variability in their triage decisions for presentations that were more appropriate for urgent or outpatient care. Among the internal medicine providers, the majority of the variability in their triage decisions stemmed from select providers ascribing to a generally more conservative triage approach. The variability in triage decisions between the physician assistant and the physicians was largely because the physician assistant did not develop the same rigorous differential diagnoses as the physicians. Such variability among individual providers may reflect biases and cognitive errors, mimic current real-life medical practice, and demonstrate the potential benefit of relying on AI for triage decisions. The variability in decisions made by individuals also demonstrates the limitation in using one human individual to determine an AI-based application’s decision accuracy, as done in previous research [11]. A strength of the current study is the utilization of a consensus decision from multiple physicians to compare and determine the accuracy of the AI-based application’s decisions.

Our findings have significant implications. The utilization of AI-based applications that improve the appropriateness and safety of medical triage has the potential to improve patient outcomes and experience as well as the efficiency of healthcare delivery. Payors, providers, and patients may benefit from cost savings and higher-value care. AI-based applications may also be able to provide triage assistance in more rural or underserved areas where access to traditional triage nursing services may be limited.

This study has limitations. We utilized clinical vignettes that were developed by a team of clinicians; therefore, limiting the real-world implications of our study. Real patients’ interaction with the AI-based application may not generate the same triage decision as for a clinical vignette simulating the same presentation of symptoms. Future research in which the AI application faces real patients is needed to ensure the accurate triage decisions seen in the present study translate to a real-world setting.

Further research with well-designed randomized trials should also evaluate the potential real-world outcomes of the utilization of AI-based triage decision support. Outcomes such as emergency department visit rates, patient morbidity and mortality, and costs may be compared for groups of patients with and without access to AI-based applications.

## Conclusions

Our study demonstrates that an AI-based application may provide accurate medical triage decision support for patients. The utilization of AI-based triage decision support may potentially improve patient outcomes and reduce healthcare costs, and these are claims that still need to be evaluated with future research.

## Appendices

The full list of clinical vignettes is listed below.

1. An 80-year-old man with a history of coronary artery disease presented with shortness of breath for two to three weeks. He complains of waking up in the middle of the night, gasping for air. He also feels uncomfortable sleeping flat, without pillows. All the symptoms started a few weeks ago.

2. A 28-year-old female with a mouth ulcer for three days. No other symptoms and no risk factors for oral cancer.

3. A 70-year-old woman with dysuria and urinary frequency for three days. She also had fevers at home for two days.

4. A 40-year-old man with weight loss and generalized itching for two months. On further questioning, he noted to have night sweats for two months as well.

5. A 30-year-old female presents with dysmenorrhea and menorrhagia for six to seven months. She also complains of feeling quite tired for about three months.

6. A 42-year-old female with hand pain and stiffness for five months. She denied any other symptoms.

7. A 28-year-old woman with a history of asthma presented with worsening of shortness of breath for a week. She had tried rescue inhalers, without much improvement. She says she has been coughing a lot for the last five days. No fever or chills.

8. A 60-year-old man who underwent hip surgery recently came with blood in phlegm for two days. He also feels shortness of breath for the last three days. His one leg appears to be a bit more swollen than the other for three days as well.

9. A 20-year-old man with sneezing and cough for two days. No fever, neck pain, or sputum production.

10. A 68-year-old man with the difficulty of swallowing for five months. He feels that the food is getting stuck at the level of the stomach and the symptom is worse with solid food. He also complained of weight loss for four to five months.

11. A 30-year-old woman presents with joint pain and a rash on the face for two months. The rash appears on both cheeks and on top of the nose. She feels very tired as well for the same duration.

12. A 35-year-old man complains of gaining weight and constipation for nine months. He also said yes to feeling tired for the last several months.

13. A 24-year-old girl who was prescribed a new pill for acne presented with shortness of breath, lip and tongue swelling, and wheezing for a few hours.

14. A 72-year-old man with fever and chills for five days. He has had a productive cough and fatigue for a week. He has a history of myocardial infarction (MI) with stents placed a few years ago.

15. A 25-year-old female with abdominal bloating for three hours. She denied any other symptoms. No weight loss or early satiety.

16. A 45-year-old man with a history of heavy smoking presents with leg swelling for two months. The pain is worse on walking and gets better with rest. He denied any other symptoms.

17. A 35-year-old obese woman presented with fatigue for six months. Upon further questioning, she attributes it to lack of sleep. Her husband admits that she snores loudly for the last several years.

18. A 28-year-old lady with a history of depression presented with feeling hopeless and tearful for two weeks. She admits feeling suicidal.

19. A 56-year-old Filipino immigrant presented with cough for two months and weight loss for six weeks.

20. A 65-year-old man with a history of MI presented with chest pain for three hours. He described the pain as a pressure-like pain and feels like an elephant sitting on the chest. He also had profuse sweating.

21. A 46-year-old woman with a breast lump and swelling in the axilla for four months. She says the breast lump is irregular in its edges.

22. A 50-year-old woman with indigestion and abdominal pain for three days. The pain is located in the right upper region. She also feels nauseous after eating for the last couple of months.

23. A 75-year-old woman who is getting treated with lung cancer, presented with swelling of her right leg for six days. The leg appears to be red and tender to touch.

24. A 72-year-old man with blood in vomitus for two days. He also complains of black stool for the same duration. He feels dizzy. He admits taking pain medications, nonsteroidal anti-inflammatory drugs (NSAIDs), almost every day for knee osteoarthritis.

25. A 40-year-old diabetic man presented with redness of the leg, associated with tenderness for seven days. He also said yes to feeling feverish and denied any other symptoms.

26. A 35-year-old man was presented with epigastric pain for two weeks. He admits taking pain medications, NSAIDs, for occasional headaches. He also feels nauseous for one week. No chest pain, shortness of breath, or diaphoresis.

27. A 34-year-old Indian engineer presented with confusion and fever for three days. He had chills and diarrhea at home. His wife said he was also nauseated for a week after a recent trip to India.

28. A 67-year-old woman presented with palpitations for a day. She feels like her heart is racing with irregular beats. She has a history of atrial fibrillation and takes warfarin at home. She also complains of feeling short of breath for a day.

29. A 42-year-old female with increased thirst and increased frequency of urination for three months. On further questioning, she complained of losing weight for two months.

30. A 75-year-old obese man presented with knee pain on both sides for six months. It causes difficulty in ambulation. He denied any other symptoms.

31. A 25-year-old man with severe shortness of breath for a few hours. The symptoms started after a motor vehicle accident in which he was the driver of the car involved. He also complains of chest pain.

32. A 50-year-old woman with a deviation of angle of mouth for two hours. She denied any muscle weakness or sensory symptoms. She also has difficulty speaking for an hour. It was noticed by her coworkers.

33. A 55-year-old man with facial pain and sinus tenderness for one month. He also has rhinorrhea for a month as well. Denied other symptoms.

34. A 38-year-old female presents with vaginal discharge and lower abdominal pain for one week. She also complained of chills for three to four days. Denied any other symptoms.

35. A 70-year-old woman with no significant past medical history presents with arm pain and upper back pain for one day. She denied any other symptoms.

36. A 40-year-old man with a history of high-risk sexual behavior presents with lumps all over his body that appear like lymph node enlargement. He also complains of losing weight. All the symptoms started a month ago.

37. A 38-year-old man with decreased hearing and ear fullness for two days. No ear pain or discharge. No fever or chills.

38. A 75-year-old woman with lower back pain for six months. Denied any red flag symptoms or steroid intake.

39. A 60-year-old immigrant with a history of heavy alcohol use in the past, presented with fatigue and abdominal distension for two months. He also noted that his eyes have been yellow for the last two months.

40. A 65-year-old man presented with fever for five days. He also has cough for five days, shortness of breath for four days, and chest pain for three days. He feels that the chest pain is worse on breathing. Fever was as high as 102 F.

41. A 29-year-old man with red-eye and eye discharge for three days. Eye discharge is watery and mild. No eye pain or other visual symptoms.

42. A 35-year-old woman with red-eye and eye discharge for four days. Red-eye is associated with moderate eye pain. Eye discharge is pus-like and significant. She also noticed blurring of vision.

43. A 65-year-old woman presenting with dyspareunia and vaginal dryness for three to four months. No history of marital conflict or depression.

44. A 29-year-old man with a history of migraine presented with severe disabling headache and photophobia for three days. The episode feels like his migraine attack. Denied fever or neck pain.

45. A 78-year-old woman with jaundice and fatigue for four months. She also complained of an abdominal lump and weight loss for three months.

46. An 84-year-old woman presented with rectal bleeding for four days. She has abdominal pain, located in the right lower region and low-grade fever at home for one week. She denied any other symptoms.

47. A 38-year-old man with palpitations and chronic diarrhea for four months. On further questioning, he said yes to weight loss for four months as well.

48. A 76-year-old woman with a history of smoking presented with a worsening of wheezing for four days. She has a history of chronic obstructive pulmonary disease and gets admitted with a change in weather.

49. A 30-year-old man with diarrhea for six months. It is mixed with mucus at times. He also complained of joint pain in multiple joints for four months.

50. A 46-year-old man with a history of uncontrolled blood presented with sudden onset of severe chest pain for two hours. Upon questioning, he felt a tearing sensation in his chest. He is very uncomfortable at rest.

The following table presents the decisions of individual providers for all 50 vignettes.

**Table 2:** Triage decisions of individual providers. ER - Emergency room MD - Medical doctor PA - Physician assistant UM - University of Michigan E - Emergency room U - Urgent care P - Primary care S - Self-care

Case	Utah ER MD	Utah ER PA	UM ER 1	UM ER 1	UM ER 3	UM ER 4	UM ER 5	UM ER 6	UCLA 1	UCLA 2	UCLA 3	UCLA 4	UCLA 5
1	E	P	E	E	E	E	E	E	E	E	E	E	U
2	S	S							S	S	S	S	S
3	E	U	E	U	U	U	U	U	U	U	E	U	U
4	P	P							P	P	U	P	P
5	P	P							P	P	P	P	U
6	P	P							P	P	P	P	P
7	E	P	E	E	P	E	E	E	U	U	U	E	U
8	E	E							E	E	E	E	E
9	S	S							S	P	S	S	U
10	P	P							P	P	P	P	P
11	P	P							P	P	P	P	P
12	P	P							P	P	P	P	P
13	E	E							E	E	E	E	E
14	E	U	E	E	E	U	E	E	E	E	E	U	U
15	S	S							S	U	P	S	S
16	E	U	E	P	P	E	U	P	P	U	P	P	U
17	P	P							P	P	P	P	P
18	E	U	E	E	E	E	E	E	E	E	E	E	E
19	P	P	E	P	P	E	P	E	E	U	E	P	U
20	E	E							E	E	E	E	E
21	P	P							P	P	P	P	P
22	U	U	E	E	E	E	U	E	E	E	E	P	U
23	E	U	E	E	E	E	U	E	E	U	E	E	U
24	E	U	E	E	E	E	E	E	E	E	E	E	E
25	E	P	E	U	E	E	U	U	U	E	E	U	U
26	P	P	E	E	E	E	E	E	U	P	E	P	U
27	E	P	E	E	E	E	E	E	E	E	E	E	U
28	E	U	E	E	E	E	E	E	E	E	E	E	E
29	P	U	U	P	P	P	U	P	P	P	P	P	U
30	P	P							P	P	P	P	P
31	E	E							E	E	E	E	E
32	E	E							E	E	E	E	E
33	U	P	P	P	P	P	P	P	P	P	P	P	U
34	U	P	U	U	U	U	U	U	U	U	E	U	U
35	E	U	E	E	E	E	U	E	E	S	E	S	U
36	P	P							E	P	P	P	U
37	P	P							U	U	U	U	U
38	U	P	P	P	P	P	P	P	P	P	P	P	P
39	U	P	P	U	P	P	U	P	P	P	E	P	U
40	E	U	E	E	E	E	E	E	E	E	E	E	U
41	P	S	S	P	P	S	S	S	U	U	P	S	U
42	E	P	E	U	U	E	U	U	E	E	U	E	U
43	P	P							P	P	P	P	P
44	P	U	U	U	U	U	U	P	U	U	U	S	U
45	P	P							P	P	E	P	P
46	U	U	E	E	E	E	E	E	E	E	E	E	U
47	P	P							E	P	P	P	U
48	E	P	U	E	U	E	E	U	U	E	E	U	U
49	P	P							P	P	P	P	P
50	E	E							E	E	E	E	E

## References

[1] Dugas AF, Kirsch TD, Toerper M (2016). An electronic emergency triage system to improve patient distribution by critical outcomes. J Emerg Med.

[2] Harding KE, Taylor NF, Leggat SG (2011). Do triage systems in healthcare improve patient flow? A systematic review of the literature. Aust Health Rev.

[3] (2021). The high cost of avoidable hospital emergency department visits. https://www.unitedhealthgroup.com/newsroom/posts/2019-07-22-high-cost-emergency-department-visits.html.

[4] Lin SY, Mahoney MR, Sinsky CA (2019). Ten ways artificial intelligence will transform primary care. J Gen Intern Med.

[5] McCall B (2020). COVID-19 and artificial intelligence: protecting health-care workers and curbing the spread. Lancet Digit Health.

[6] Davenport T, Kalakota R (2019). The potential for artificial intelligence in healthcare. Future Healthc J.

[7] Raita Y, Goto T, Faridi MK, Brown DF, Camargo CA Jr, Hasegawa K (2019). Emergency department triage prediction of clinical outcomes using machine learning models. Crit Care.

[8] Chambers D, Cantrell AJ, Johnson M, Preston L, Baxter SK, Booth A, Turner J (2019). Digital and online symptom checkers and health assessment/triage services for urgent health problems: systematic review. BMJ Open.

[9] Hill MG, Sim M, Mills B (2020). The quality of diagnosis and triage advice provided by free online symptom checkers and apps in Australia. Med J Aust.

[10] Semigran HL, Linder JA, Gidengil C, Mehrotra A (2015). Evaluation of symptom checkers for self diagnosis and triage: audit study. BMJ.

[11] Baker A, Perov Y, Middleton K (2020). A comparison of artificial intelligence and human doctors for the purpose of triage and diagnosis. Front Artif Intell.

[12] Entezarjou A, Bonamy AE, Benjaminsson S, Herman P, Midlöv P (2020). Human- versus machine learning-based triage using digitalized patient histories in primary care: comparative study. JMIR Med Inform.

[13] Sadeghi S, Barzi A, Sadeghi N, King B (2006). A Bayesian model for triage decision support. Int J Med Inform.

[14] Dang J, Okurowski E, Gelburd R, Limpahan L, Iny N (2015). Urgent care facilities: geographic variation in utilization and charges for common lab tests, office visits, and flu vaccines. Conn Med.

[15] Barriga EM, Ferrer IP, Sánchez MS, Baranera MM, Utset JM (2017). A new artificial intelligence tool for assessing symptoms in patients seeking emergency department care: the Mediktor application. (Article in Spanish). Emergencias.

[16] Gilbert S, Mehl A, Baluch A (2020). How accurate are digital symptom assessment apps for suggesting conditions and urgency advice? A clinical vignettes comparison to GPs. BMJ Open.

